# Enacting Social Justice Leadership: Principals’ Modes of Involvement in Urban Secondary Schools in Canada

**DOI:** 10.1177/00131245261492662

**Published:** 2026-09-28

**Authors:** Manu Sharma

**Affiliations:** 1 Thompson Rivers University, Kamloops, BC, Canada

**Keywords:** principal, Canada, urban, social justice initiatives, secondary schools

## Abstract

This qualitative study examines how principals enact social justice through different modes of leadership involvement in urban Canadian secondary schools. Drawing on semi-structured interviews with principals and vice-principals across three Canadian cities and contextual analysis of school-based initiatives, the study develops an empirically grounded typology of leadership enactment. The findings identify three distinct but interrelated modes of involvement: peripheral support and legitimation, community-embedded brokerage, and structural and curricular intervention. Instead of presenting social justice leadership as a singular or idealized practice, the typology demonstrates how principals’ involvement varies in visibility, authority, and risk, shaped by institutional, political, and geographic conditions. By shifting attention from leaders’ commitments or dispositions toward leadership enactment, this study extends scholarship on educational administration and social justice leadership. The typology offers a conceptual framework for examining how equity-oriented initiatives emerge, are sustained, and remain vulnerable within public school systems, with implications for leadership practice, preparation, and future research.

## Introduction

In recent years, social justice initiatives in schools have become increasingly visible and politically contested, particularly as debates over equity, diversity, and inclusion intensify across public institutions. Principals are often positioned at the intersection of these debates, expected to respond to inequities while navigating policy mandates, accountability structures, and community pressures. These conditions raise important questions about how social justice leadership is enacted in practice, and what forms of involvement are possible or constrained within contemporary school systems.

Social justice has become a central yet contested priority in public education, particularly in urban school systems marked by persistent inequities related to race, class, gender, and ability. While schools are increasingly expected to address these inequities, principals must operate within political, institutional, and community contexts that both enable and constrain social justice efforts. Existing scholarship on social justice leadership has documented principals’ commitments, beliefs, and moral orientations toward equity-oriented work (Ayanoğlu & Arastaman, 2023; Cambron-McCabe & McCarthy, 2005; Moral Santaella, 2022; Shields & Hesbol, 2020; Wang, 2018). This literature has made important contributions by highlighting leadership dispositions, values, and advocacy roles. However, less attention has been paid to *how* principals enact social justice in practice, particularly through their varying degrees of involvement in initiatives that aim to address inequity within schools.

Much of the literature treats social justice leadership as a coherent or unified practice, often emphasizing what principals *should* do instead of examining the diverse ways they engage in social justice work under different conditions. As a result, we know relatively little about how principals’ roles vary in relation to social justice initiatives, or how contextual factors shape these variations.

Leadership involvement in social justice initiatives can take multiple forms, ranging from symbolic support and facilitation to direct intervention in curriculum, policy, and organizational structures. Attending to these differences matters because leadership involvement shapes not only how initiatives are implemented, but also their visibility, sustainability, and vulnerability to political or institutional change. Yet, these distinctions are rarely examined explicitly in studies of social justice leadership.

This study addresses this gap by examining how principals enact social justice through different modes of leadership involvement in urban Canadian secondary schools. Drawing on qualitative data from interviews with principals and an analysis of school-based initiatives, the study asks: *How do principals enact social justice through different modes of leadership involvement in urban Canadian secondary schools?*

To examine how principals engage in social justice work under these conditions, this study adopts a theoretical and conceptual framework that brings together social justice leadership and leadership enactment. This framework allows for analytic attention to both the normative commitments associated with equity-oriented leadership and the practical ways leadership is carried out within institutional constraints.

It is important to note that in this study, social justice work is understood as praxis, an ongoing process of reflection and action aimed at challenging inequities and transforming schools into more just and equitable institutions (Furman, 2012). In educational leadership contexts, this involves making issues of race, class, gender, disability, and other forms of marginalization central to leadership practice, decision-making, and advocacy (Theoharis, 2007). Moreover, that social justice work done critically involves engaging with the structural conditions of power that shape how inequities are produced, maintained, and contested within educational institutions (Young, 1990).

In this study, the term urban is used to refer not simply to population size or density, but to school contexts situated within large metropolitan areas characterized by demographic diversity, institutional complexity, and varied social conditions. The urban schools in this study—located in Montreal, Toronto, and Vancouver—serve student populations shaped by diverse and intersecting identities, including but not limited to race, culture, language, gender, socioeconomic status, and other forms of social difference. These schools operate within policy and community environments influenced by broader political, economic, and geographic factors. This understanding aligns with scholarship that conceptualizes urban schooling as shaped by intersecting conditions of diversity, inequality, and institutional constraint rather than as a singular or deficit-based category (Milner, 2012; Paris & Alim, 2017).

With the above in mind, this article situates the study within literature on principals and social justice leadership in urban schools. It then outlines the research design and analytic approach. The findings section presents the typology of principal involvement, followed by a discussion of its implications for leadership practice, preparation, and research.

## Theoretical and Conceptual Framework

This study draws on a composite, practice-based framework that integrates social justice leadership and leadership enactment to examine how equity-oriented leadership is carried out in practice. Social justice leadership in education foregrounds educational leaders’ ethical responsibility to inclusion, diversity, and equity, and for disrupting inequities related to race, class, gender, and other forms of marginalization within schools (Ayanoğlu & Arastaman, 2023; Beycioğlu et al., 2018; Flores & Kyere, 2021; Theoharis, 2008; Zachos & Mandala, 2024). Scholars in this tradition emphasize the central role of principals in shaping school cultures that promote inclusion, equity, and democratic participation, often within contexts marked by political contestation and resistance. Thus, these scholars articulate the moral and normative commitments that undergird leadership for social justice.

At the same time, scholars have noted that social justice leadership research can at times privilege leaders’ commitments, dispositions, or advocacy while paying less sustained attention to how equity-oriented leadership is enacted within complex organizational, community, and political conditions (Shields, 2010; Theoharis, 2007). To address this limitation, the present study draws on practice-oriented and leadership-as-practice theories of leadership enactment (Raelin, 2011; Vennebo, 2017), which conceptualize leadership not as an individual attribute or formal role, but as a set of situated practices shaped by context and authority. Leadership enactment is the process of translating leadership theories, values, and policies into tangible actions and behaviors in a specific social or professional context. It shifts the focus from leadership as a static position to leadership as a dynamic, relational performance. Leadership as practice focuses on the everyday practice of leadership including its moral, emotional, and relational aspects, rather than its rational, objective, and technical aspects. It looks for leadership in its activity as opposed to through the traits and heroics of individual actors. Leadership-as-practice is concerned about where, how, and why leadership work is being organized and accomplished than about who is offering visions for others to do the work.

Collectively these frameworks enable an examination of social justice leadership as enacted practice in contrast to an ideology or disposition alone. While social justice leadership provides the normative orientation clarifying *why* equity-oriented leadership matters, leadership enactment offers an analytic lens for examining *how* principals take up, negotiate, and perform this work in contextually specific ways. Importantly, this study does not position social justice leadership or leadership enactment as standalone grand theories; instead, they are mobilized as complementary, theory-informed lenses that together support a practice-based analysis of leadership. This integrated framework foregrounds variation, context, and constraint, making it possible to analyze differences in principals’ modes of involvement without presuming a singular or idealized model of leadership. In doing so, the framework supports the development of an empirically grounded typology that captures how social justice leadership is enacted across diverse institutional and political contexts.

Consistent with this integrated, practice-based framework, the findings identified three distinct but overlapping modes through which principals enacted social justice leadership. The first mode identified in the findings, peripheral support and legitimation, reflects an indirect form of leadership in which principals authorize, protect, and sustain equity-oriented work while allowing students and staff to retain primary ownership. The second mode, community-embedded brokerage, captures a relational and outward-facing form of enactment in which principals connect schools and students with community resources, partnerships, and opportunities. The third mode, structural and curricular intervention, represents a more direct and institutionally embedded form of enactment in which principals use formal authority to reshape curriculum, professional learning, and organizational structures in pursuit of equity. These modes emerged inductively from the analysis rather than functioning as predetermined theoretical categories, while the integrated framework provides the conceptual basis for interpreting variation in leadership authority, visibility, context, and constraint.

## Literature Review: Principals and Social Justice Leadership in Urban Schools

Scholarship on social justice leadership has established that principals play a critical role in advancing equity and inclusion, particularly in schools serving diverse and historically marginalized student populations. A dominant theme across the literature is the framing of social justice leadership as a moral or ethical commitment. Studies portray principals as values-driven actors who advocate for equity and seek to challenge inequitable structures (Brooks et al., 2007; Theoharis, 2008). At the same time, other work highlights the ways in which these commitments are enacted through specific practices, such as community engagement, resource redistribution, and organizational change (DeMatthews & Mawhinney, 2014; Merchant & Garza, 2015). While this body of research has been instrumental in legitimizing social justice as a central concern of educational leadership, it has paid comparatively less attention to variation in how principals enact these commitments under different contextual conditions.

### Urban Contexts: Relevance Without Explicit Theorization

Although social justice leadership research frequently references diversity, marginalization, and inequality conditions often associated with urban schooling, surprisingly few studies explicitly situate their analyses within urban school sites. Across the literature reviewed, urban contexts are frequently implied rather than theorized. Principals are described as working in large, diverse, and resource-constrained settings, yet the spatial, political, and institutional dynamics of urban schooling are rarely examined as shaping leadership practice. This lack of explicit attention to urban context obscures how place-based conditions influence what forms of social justice leadership are possible, risky, or constrained.

### Principals’ Commitments and the Moral Framing of Social Justice

A dominant theme across the literature is the framing of social justice leadership as a moral or ethical commitment. Studies consistently portray principals as values-driven actors who articulate inclusive school visions, emphasize fairness and care, and seek to create positive school climates (Ayanoğlu & Arastaman, 2023; Sun, 2019; Wasonga, 2009). Social justice leadership is often associated with qualities such as persistence, relationality, inclusiveness, and advocacy, as well as the symbolic work of establishing mission statements and shared values. While this focus highlights the ethical foundations of leadership, it frequently stops short of examining how these commitments translate into concrete actions beyond vision-setting and climate-building. At the same time, this framing may obscure the ways in which principals’ engagement in social justice work is shaped by contextual constraints, institutional conditions, and strategic considerations rather than moral conviction alone.

### Structural Constraints: Accountability, Policy, and Institutional Pressure

Across the literature, a recurring and powerful theme is the tension between equity-oriented leadership and accountability regimes. Principals consistently report being caught between policy mandates, performance metrics, and social justice goals (Chaaban et al., 2025; Lopez, 2019; Wang, 2017a, 2017b). Accountability frameworks that privilege quantifiable outcomes—such as test scores or graduation rates—often limit principals’ capacity to address students’ lived realities, intersectional identities, and structural inequities. Even when equity language is embedded in policy rhetoric, implementation procedures frequently overlook race, disability, gender identity, and socioeconomic status (Campbell, 2021). As a result, principals describe a disconnect between their social justice values and the forms of leadership practice that are institutionally rewarded.

### Social Justice Leadership as Constrained and Contested Practice

Relatedly, the literature underscores the contested nature of social justice leadership in practice. Principals who seek to redistribute resources, challenge exclusionary practices, or engage communities often encounter resistance from parents, staff, district officials, and broader political environments (Kavrayici, 2024; Theoharis, 2007, 2008, 2023). Studies of principals working in urban contexts, in particular, highlight the emotional and political costs of this work, including resistance to inclusion efforts, pressures to raise test scores, and limited financial or institutional support. These findings suggest that social justice leadership is not a singular or stable practice, but one that is continually negotiated within shifting power relations.

### Identity, Sense-Making, and Leadership Decision-Making

Another cross-cutting theme concerns principals’ interpretations of student identity and how these interpretations shape leadership practice. Research demonstrates that principals’ assumptions about race, class, disability, and behavior influence their approaches to inclusion, discipline, and resource allocation (Pollock & Briscoe, 2020; Wang, 2017a). Principals are often called upon to interrogate their own beliefs and engage in ongoing sense-making as they encounter difference in their school communities. However, while these studies illuminate how identity perceptions shape leadership orientations, they provide less insight into how principals move from awareness or reflection to sustained, action-oriented social justice initiatives.

### Emphasis on Commitment Over Enactment

Taken together, the literature privileges principals’ commitments, values, and orientations toward social justice, while offering limited attention to how social justice leadership is enacted through different forms of involvement. Even studies that document activism, community engagement, or inclusive practices often describe leadership in broad terms, without detailing the specific actions principals take, the degree of their involvement, or how they strategically engage—or disengage—from equity initiatives over time (DeMatthews, 2015, 2018, 2019; Sun, 2019). As a result, leadership practice is frequently presented as either present or absent, as opposed to variable, contingent, and contextually shaped.

### Gap and Contribution

Despite a robust and growing body of scholarship on social justice leadership, there remains limited empirical attention to variation in principals’ modes and degrees of involvement in equity-oriented work, particularly within urban Canadian secondary school contexts. Existing studies tend to emphasize advocacy, resistance, or moral commitment, but do not sufficiently examine how principals lead, support, broker, legitimize, or strategically distance themselves from social justice initiatives under different institutional and political conditions.

This study addresses this gap by examining how principals enact social justice through distinct modes of leadership involvement in urban secondary schools. By shifting the analytic focus from intention to enactment, the study offers a practice-based, theory-informed account of how social justice leadership is realized and constrained in everyday school leadership.

## Research Design and Methods

This study adopts a qualitative, interpretive approach, drawing on cross-case analysis to examine how principals make sense of and enact social justice leadership in context (Merriam & Tisdell, 2016). Moreover, this qualitative study examines how principals enact social justice through their involvement in school-based initiatives in urban Canadian secondary schools. A qualitative design was selected to capture principals’ interpretations of social justice, their leadership practices, and the contextual conditions shaping their involvement.

### Site Selection and Participants

The six schools were located across three urban contexts—Montreal, Toronto, and Vancouver—each characterized by distinct geographical, political, institutional, and community conditions that shaped the enactment of social justice work. In the Montreal sites, provincial policy and public discourse placed constraints on discussions of religion and certain political issues, and both schools served lower socioeconomic communities with limited resources, shaping a focus on access-based supports. In Toronto, the schools were situated in highly diverse urban contexts and were influenced by targeted equity initiatives and leadership commitments, including programming focused on Black and Indigenous student success. In Vancouver, school contexts were shaped by ongoing public debates regarding police presence in schools, as well as strong regional attention to Indigenous sovereignty, climate change, and urban issues such as homelessness. These contextual differences informed the analysis by providing insight into the conditions under which particular forms of leadership involvement emerged. Moreover, these contextual differences were identified through participants’ accounts, supported by publicly available school materials and the researcher’s contextual knowledge of the school systems in which the study was conducted.

In addition, schools were identified based on publicly available evidence of an explicit commitment to social justice, equity, diversity, or inclusion within school plans, mission statements, or publicly described initiatives. This purposeful sampling strategy was used to examine variation in leadership enactment within contexts where social justice was already a stated priority.

The study included six school leaders working in urban secondary schools across Montreal, Toronto, and Vancouver, including four principals and two vice-principals. Participants self-identified as four women and two men, and represented diverse racial identities, including Black, South Asian, mixed-race, and White. Participants brought varied professional trajectories and levels of leadership experience. Some were in the early stages of their administrative roles, while others had over a decade of experience in educational leadership, often following prior roles as teachers or district-level staff. Participants’ tenure at their current schools ranged from 1 to 5 years. Participants were not pre-classified as “social justice leaders.” Instead, schools were purposively selected based on publicly available evidence of engagement with equity and social justice initiatives. The study then examined how principals and vice-principals within these contexts described and enacted their involvement in such work.

### Data Sources

Data were drawn from two primary sources: online zoom semi-structured interviews with school leaders and publicly available school website materials. Interviews served as the primary source of data, focusing on principals’ understandings of social justice, their roles in developing or sustaining initiatives, and the challenges and opportunities associated with this work. Website materials were used primarily to support site selection and contextual familiarization. Specifically, school websites, including mission statements, school plans, and descriptions of programs or initiatives were reviewed to identify schools that demonstrated an explicit commitment to equity, diversity, inclusion, or social justice–oriented work. These materials also provided background context regarding school priorities and publicly articulated values, which informed interpretation of interview data but were not subjected to formal document analysis.

Moreover, the key data source were online semi-structured interviews focused on principals’ understandings of social justice, their roles in developing or sustaining initiatives, and the challenges and opportunities associated with this work. Each interview lasted approximately 45 to 60 min and was audio-recorded and professionally transcribed.

### Analytic Approach

Data were analyzed using a thematic analysis approach, which involved identifying, analyzing, and interpreting patterns across participants’ accounts (Braun & Clarke, 2006). The analysis proceeded through multiple, iterative stages. First, interview transcripts were read closely and coded inductively to identify patterns related to leadership involvement, decision-making, and responsibility for social justice initiatives. Particular attention was paid to how principals described their roles, actions, and levels of engagement. Second, codes were grouped into broader analytic categories through constant comparison, capturing similarities and differences in how principals enacted social justice work. Analysis was conducted both within each site (Montreal, Toronto, and Vancouver) to identify context-specific patterns, and across sites to examine variation in leadership enactment across contexts (Merriam & Tisdell, 2016). Third, these analytic categories were synthesized into a typology of principal involvement. The resulting typology represents three distinct but overlapping modes of leadership enactment, reflecting differences in the degree, form, and visibility of principal involvement in social justice initiatives. The typology emerged inductively through iterative engagement with the data, informed by contextual materials.

Throughout the analytic process, attention was paid to contextual factors, including geographic location, political climate, and institutional conditions that shaped leadership enactment. In contrast to treating context as a background variable, the analysis considered how these conditions enabled or constrained particular modes of involvement. Several strategies were used to enhance the trustworthiness of the analysis, including member checking for participants who requested transcript review, analytic memo-writing to document interpretive decisions, and the use of multiple data sources to support analytic claims. These strategies strengthened the credibility and transparency of the analytic process.

## Findings

### Mode 1: Peripheral Support and Legitimation

In the first mode of involvement, principals enact social justice through peripheral support and legitimation. While this mode was most clearly articulated in Ali’s account, elements of indirect involvement and institutional support were also evident, to varying degrees, across other participants’ descriptions of leadership practice. In this mode principals position themselves as facilitators who authorize, protect, and sustain social justice work initiated by students or teachers. Consequently, leadership in this mode is enacted through strategic restraint, where principals use their positional authority to create conditions for equity-oriented work without assuming ownership of its content or direction. Principals operating in this mode emphasize distributed leadership, teacher autonomy, and the importance of student- and staff-led engagement with social justice issues. Their involvement is intentional but indirect: they focus on ensuring that those leading difficult conversations feel institutionally supported, particularly when such conversations involve risk, discomfort, or conflict.

As Vancouver principal Ali explained, teachers must feel protected when engaging in social justice work, “[t]eachers also need to feel like they are going to be supported if a conversation goes sideways or if it gets uncomfortable or if it gets heated.” As a opposed to positioning himself as the primary leader of these conversations, this principal described his role as creating supportive structures around them, including regular leadership team meetings where issues of racism and equity could be discussed collectively,

[w]e meet as a school leadership team once a month, and one of the recurring themes that is going to be taking place from this point forward will be the issue of racism and basically how we can support colleagues in those conversations.

Here, leadership is enacted not through directive intervention but through institutional legitimation—signaling that equity-focused work is recognized, protected, and worthy of sustained attention.

Peripheral support was also evident in how principals described coordinating institutional resources to support marginalized students while maintaining a collaborative leadership stance. For example, principal Ali outlined how support for Indigenous students was structured through dedicated roles and collective responsibility:

We have two dedicated staff members—an Indigenous resource teacher and an Indigenous support worker—to liaise with families. And when I talk about the ‘school team,’ that includes administration, counsellors, resource staff, and classroom teachers—we come together to speak specifically about the needs of our Indigenous students.

In this example, the principal does not present himself as the architect of the initiative but as a convenor who ensures that institutional supports are aligned and that responsibility is shared across roles. This mode of involvement was most evident in Vancouver-based schools, where principals appeared attentive to balancing commitments to social justice with broader political sensitivities and governance expectations. In these contexts, peripheral support allowed principals to advance equity-oriented work while minimizing the risks associated with overt administrative leadership in contested areas. Such an approach reflects leadership shaped by institutional caution: principals remain visibly supportive of social justice initiatives while allowing teachers and students to maintain primary ownership of the work.

However, while peripheral support and legitimation can foster grassroots engagement and protect teacher autonomy, this mode also carries limitations. Because initiatives are not structurally embedded or formally led by administration, their sustainability may depend heavily on individual staff members or student leaders. As a result, equity-oriented work enacted through this mode can remain vulnerable to staff turnover, shifting priorities, or changes in school leadership. Importantly, although this mode may appear limited in terms of direct engagement, it reflects how principals navigate contextual constraints, including competing priorities, institutional expectations, and political sensitivities. Peripheral support and legitimation can play a meaningful role in enabling social justice initiatives to emerge and persist, particularly in contexts where more direct or visible leadership may carry professional or political risk. At the same time, this mode raises important questions about the extent to which such approaches may inadvertently defer responsibility or limit the transformative potential of social justice work. Thus, the first mode reflects leadership enacted through restraint and institutional legitimation, while the second mode involves more outward-facing engagement with community-based resources.

### Mode 2: Community-Embedded Brokerage

In the second mode of involvement, principals enact social justice through community-embedded brokerage. In this mode, principals position themselves as connectors between the school and external community resources, leveraging partnerships, programs, and networks to expand students’ access to social, emotional, and economic supports. Leadership enactment here is relational and outward-facing, with principals mediating between institutional structures and community-based opportunities.

Principals operating in this mode understand social justice as deeply connected to students’ well-being, belonging, and access to opportunities beyond the classroom. As an alternative to focusing primarily on curricular or policy reform, these principals emphasize programs that address students’ holistic needs—mental health, identity development, and pathways to work or further education. Leadership involvement is direct but facilitative: principals initiate, sustain, and normalize programs that connect students with community-based supports, while allowing those programs to function as integrated parts of the school environment rather than as add-on initiatives.

This second mode was most evident in the Montreal context, although aspects of this form of leadership were also present across other settings where principals described drawing on community-based resources to support students. Principal Olu described a school-based program focused on students’ emotional and mental well-being, “[w]e have a program called LIFT that basically . . . it deals with yoga and mindfulness, and the kids are able to discuss a lot of the issues that teenagers go through on a daily basis.” Thus, social justice here is enacted through attention to students’ emotional lives and everyday stressors, recognizing well-being as a prerequisite for meaningful engagement with schooling.

The same principal also emphasized programs designed to connect students with community-based economic opportunities, “[w]e have a program called the Student Business Program, which gives kids access to internships in different types of jobs in community. So, since I started here, we’ve been doing that program. It’s been very, very, very successful.”

In this example, Principal Olu acts as a broker, facilitating access to internships and community partnerships that extend students’ learning beyond the school and into real-world contexts.

Importantly, he articulated a clear theory of change linking participation in these programs to longer-term educational outcomes:

If they know they have this club on one day—they’re coming in and they’re participating with their classmates—it makes them feel stronger in terms of their sense of belonging to the school, which in turn helps them feel more confident in terms of their schoolwork, which in turn helps them to graduate, which in turn helps them if they decide to go to their college or trade school.

This account highlights how community-embedded initiatives are understood not as isolated supports, but as part of a broader ecosystem that fosters belonging, persistence, and post-secondary pathways.

Another example of community-embedded brokerage was evident in how principals cultivated reciprocal relationships with local businesses and organizations. Instead of positioning community partnerships as one-directional supports for students, principals described these relationships as mutually beneficial and sustained through shared investment.

For example, Leonardo a Montreal principal explained, “[a] lot of these community and business people will come here and partner with us just to get some exposure to the students—as a company that likes to invest in its youth kind of thing.” This account highlights the principal’s role as an intermediary who aligns the interests of community partners with the school’s social justice goals. Through such brokerage, principals create opportunities for students while simultaneously positioning the school as a valued community institution. Social justice leadership in this mode is enacted through relationship-building that expands students’ access to social and economic capital without requiring formal policy change or curricular restructuring.

Thus, community-embedded brokerage emerged most strongly in contexts where principals faced political or institutional constraints on overt curricular or policy-level intervention. In such settings, community partnerships offered a viable and socially acceptable pathway for addressing inequities related to socioeconomic status, student well-being, and access to opportunity.

This mode reflects a leadership strategy shaped by contextual pragmatism: principals mobilize external resources to compensate for institutional limitations while maintaining alignment with broader governance expectations.

Nevertheless, while community-embedded brokerage can meaningfully expand students’ access to supports and opportunities, it also presents limitations. Programs dependent on external partnerships may be vulnerable to funding changes, shifting community relationships, or leadership turnover. Additionally, because these initiatives often operate alongside instead of within formal curricular structures, their impact may remain uneven across student populations.

In contrast to the facilitative and brokerage-oriented approaches described above, the third mode represents the most direct and institutionally embedded form of leadership involvement.

### Mode 3: Structural and Curricular Intervention

In the third mode of involvement, principals enact social justice through structural and curricular intervention. In this mode, principals use their formal authority to initiate, implement, and sustain changes to school structures, curriculum pathways, and professional learning practices. Leadership enactment here is direct and visible, positioning social justice as an institutional priority instead of as an optional or peripheral initiative.

Principals operating in this mode understand social justice as requiring systemic change within the organization of schooling itself. As opposed to relying on primarily grassroots initiatives or external partnerships, these principals intervene at the level of curriculum, professional development, and instructional structures. Their leadership involves deliberate agenda-setting, coordination across departments, and the strategic use of formal roles and processes to embed equity-oriented work into the everyday functioning of the school.

This mode also entails heightened political and interpersonal risk. Principals must navigate staff resistance, competing priorities, and public scrutiny while maintaining momentum for change.

Another example of community-embedded brokerage was evident in how principals cultivated reciprocal relationships with local businesses and organizations. One vice-principal, Tiara, described leading initiatives that formalized pathways for Black students, explicitly linking social justice work to post-secondary access, “[o]ne of those initiatives was, in fact, an African Heritage Committee and also working with students around pathways . . . Black students that needed pathways and were exploring pathways in college and university.” In this instance, leadership extends beyond symbolic recognition to the institutional organization of opportunity, positioning equity as a matter of access, guidance, and long-term outcomes.

Vice-Principal Tiara also described how this work became institutionally recognized and validated through formal accolades, “[t]here was the Black Heritage assembly . . . and, in fact, that committee won an award because of the tremendous work they had done in the school.”

This recognition reflects how structural initiatives, once embedded, can generate institutional legitimacy that extends beyond the school itself. In addition to student-facing initiatives, both Toronto based principals in this mode also led sustained professional learning focused explicitly on race and equity. Principal Nateesha described intentionally staging this work to manage staff readiness and resistance, “[w]e started with book clubs. That was the conduit to have the critical conversations in small group . . . Now, the fact that my staff can actually talk about white power and white privilege and not get their knickers in a twist, I’m shocked.” She further described beginning with a small, carefully selected group,“[w]e got a small group together called *So You Want to Talk About Race?*—eight staff members who were predominantly white. I was shocked.”

Both these accounts illustrate leadership as strategic containment: principals deliberately structured spaces, named initiatives cautiously, and sequenced dialogue to ensure that equity work could proceed without being derailed.

Curricular restructuring further exemplified this mode. Vice-principal Tiara described proposing and implementing a formal First Nations, Métis, and Inuit pathway across Grades 9 to 12, “[t]hat’s the one I’ve been working on for some time.” The initiative required coordination across departments and formal approval processes, underscoring the vice-principal’s direct involvement in reshaping curricular structures compared to merely supporting existing efforts. These examples illustrate that the modes are not confined to particular sites or individuals, but reflect patterns of leadership enactment that emerge differently depending on contextual conditions.

Structural and curricular intervention emerged most clearly in contexts where principals had both the positional authority and institutional mandate to initiate change. However, these contexts also involved heightened political sensitivity, requiring principals to engage in careful framing, sequencing, and coalition-building to sustain momentum. This mode reflects a leadership approach shaped by urgency and accountability: principals act directly to institutionalize social justice while managing the risks associated with visible change.

Nonetheless, while structural and curricular intervention offers the greatest potential for institutionalizing social justice, it also places principals in highly visible and vulnerable positions. These initiatives often rely on sustained administrative leadership and may face resistance from staff or community members. Without broader system-level support, such interventions risk being weakened or reversed following leadership transitions.

## Discussion

This study examined how principals enact social justice through different modes of leadership involvement in urban Canadian secondary schools. The findings show how these modes—peripheral support and legitimation, community-embedded brokerage, and structural and curricular intervention—operate as contextually responsive patterns of leadership practice.

The three modes of leadership involvement should not be understood as discrete or mutually exclusive categories, nor as points along a developmental continuum. The modes represent analytically distinct but empirically overlapping forms of leadership enactment. Principals may move between modes across different initiatives or moments, depending on contextual conditions, including institutional constraints, political climate, and community dynamics. As such, the typology is intended not to rank or stage leadership practice, but to capture variation in how social justice work is enacted in contextually situated and responsive ways. By developing a three-mode typology—peripheral support and legitimation, community-embedded brokerage, and structural and curricular intervention—the findings extend existing scholarship on social justice leadership by shifting attention from leaders’ commitments or dispositions to the *forms and conditions of leadership enactment*.

## Social Justice Leadership as Varied Enactment

Much of the literature on social justice leadership emphasizes moral purpose, advocacy, and commitment to equity. While this work has been essential in establishing the ethical foundations of leadership for social justice, it often implies a singular or idealized model of practice. The typology presented here complicates this assumption by demonstrating that principals enact social justice through distinct and contextually situated modes of involvement, as reflected in the patterns identified across the three modes in the findings.

As an alternative to representing a linear progression or hierarchy, the three modes reflect different leadership logics shaped by institutional constraints, political climates, and organizational cultures. Principals in this study did not simply choose how to engage in social justice work based on personal preference; alternatively, their involvement was mediated by the risks and possibilities present in their specific contexts. This finding underscores the importance of understanding social justice leadership as situated practice, rather than as a fixed set of behaviors.

## Leadership, Risk, and Institutional Constraint

Across all three modes identified in the findings, leadership enactment was closely tied to principals’ assessments of institutional and political risk. In the mode of peripheral support and legitimation (Mode 1), principals enabled equity-oriented work by authorizing and protecting teacher- and student-led initiatives while limiting their own visibility. This approach allowed social justice work to proceed in contexts where overt administrative leadership might provoke resistance or jeopardize trust.

Community-embedded brokerage (Mode 2) similarly reflected a pragmatic leadership response to constraint. By leveraging partnerships with external organizations and community actors, principals expanded students’ access to social, emotional, and economic resources without directly altering formal curricular or policy structures. In these cases, social justice leadership was enacted through relational mediation instead of institutional reform.

Structural and curricular intervention (Mode 3) represented the most visible and high-risk mode of involvement. Principals operating in this mode used formal authority to reshape curriculum pathways, professional learning structures, and organizational priorities. However, these interventions required careful sequencing, strategic naming, and ongoing negotiation with staff and stakeholders. The findings suggest that structural leadership for social justice is not simply a matter of decisiveness or courage, but of strategic navigation within contested institutional spaces and power. The findings point to the importance of understanding social justice leadership in relation to systems of power. While this study focuses on leadership enactment, the modes identified reflect how principals navigate and respond to institutional structures that shape participation, access, and opportunity. As Young (1990) suggests, inequities are produced through structural relations rather than isolated actions, and the variation across modes illustrates how principals engage these conditions in different ways—whether by working within constraints, leveraging external resources, or intervening directly in institutional practices.

## Implications for Sustainability and Leadership Transitions

The typology also reveals important implications for the sustainability of social justice initiatives. While structural and curricular interventions offer the greatest potential for institutionalization, they are often closely tied to individual leaders’ authority and advocacy. Conversely, peripheral and brokerage-oriented approaches may foster broader participation but risk remaining informal or unevenly distributed.

Thus, the findings raise critical questions about leadership transitions and continuity. Social justice initiatives that depend heavily on individual principals—regardless of mode—may be vulnerable to erosion when leadership changes. This suggests a need for greater attention to how social justice leadership can be collectively embedded within schools and districts, as opposed to residing solely in individual leadership practices.

## Implications for School Leadership Practice

The typology developed in this study offers practical insight into how principals engage in social justice work within the constraints of everyday school leadership. The findings suggest that principals enact social justice through different modes of involvement depending on context, institutional conditions, and perceived risk. Importantly, these modes should not be understood hierarchically. Each represents a legitimate form of leadership practice that may be appropriate in particular organizational or political environments.

For practicing principals, the typology provides a reflective tool for examining their own leadership involvement in equity-oriented initiatives. Principals may recognize moments in which they deliberately adopt a peripheral role to legitimate and protect teacher- or student-led work, particularly when direct administrative leadership could provoke resistance or undermine trust. In other contexts, principals may enact social justice through community-embedded brokerage, leveraging partnerships and external resources to expand students’ access to social, emotional, or economic supports. In schools where institutional conditions allow, principals may engage more directly through structural and curricular interventions that embed social justice within formal organizational practices.

The findings also highlight that principals may move between modes over time or across initiatives. Leadership involvement is not fixed, but responsive to changing conditions, including staff readiness, community expectations, and policy pressures. Recognizing this fluidity can help principals avoid deficit interpretations of their own leadership, particularly when they are unable to engage in more visible or structural forms of social justice work.

For leadership preparation and professional learning, this study underscores the importance of preparing principals to navigate complexity in contrast to adopting prescriptive models of social justice leadership. Preparation programs may benefit from engaging aspiring leaders in case-based discussions that examine how different modes of involvement operate in practice, the trade-offs associated with each, and the strategies principals use to assess risk and sustain equity-oriented work. By foregrounding leadership enactment as opposed to idealized dispositions, the typology supports a more realistic and context-sensitive understanding of social justice leadership in schools.

## Contributions to School Leadership Research

This study contributes to school leadership research in three keyways. First, it offers an empirically grounded typology that clarifies how principals enact social justice through varying forms of involvement, moving beyond binary distinctions between active and passive leadership. Second, it foregrounds the role of contextual constraint in shaping leadership practice, demonstrating that principals’ engagement in social justice work is deeply influenced by institutional, political, and organizational conditions. Third, it reframes social justice leadership as a matter of enactment and professional responsibility instead of solely as individual moral orientation.

By foregrounding variation, risk, and context, this study provides a framework for understanding how principals navigate equity-oriented work in their daily leadership practice. This research encourages school leadership scholars and practitioners to examine how leaders assess context, manage competing demands, and enact social justice initiatives within the constraints they face.

## Conclusion

This study examined how principals enact social justice through different modes of leadership involvement in urban Canadian secondary schools. By developing a three-mode typology—peripheral support and legitimation, community-embedded brokerage, and structural and curricular intervention—the findings provide insight into how principals engage in equity-oriented work under varying institutional and political conditions.

In place of positioning social justice leadership as a singular or idealized practice, the findings demonstrate that principals engage in equity-focused initiatives through multiple, contextually shaped forms of involvement. These modes reflect differences not only in leadership approach, but also in how principals assess risk, exercise authority, and take responsibility within their schools. In this way, the study shifts attention from leaders’ stated commitments to the everyday decisions and actions through which social justice work is carried out.

The findings also underscore the contingent nature of social justice initiatives in public schooling. Across all three modes, principals’ leadership involvement was shaped by broader contextual forces, including governance structures, community expectations, and political sensitivities. While some modes enabled greater institutionalization of equity-oriented practices, others supported broader participation or reduced administrative visibility. Each mode involved distinct possibilities and trade-offs, reinforcing the importance of understanding how context shapes leadership practice.

This study contributes to school leadership research by offering an empirically grounded framework for examining how principals enact social justice in practice. By foregrounding variation, context, and leadership judgment, the typology provides a useful lens for scholars and practitioners seeking to understand how equity-oriented initiatives are initiated, supported, and sustained in schools. In contrast to prescribing a single model of effective leadership, the framework invites closer attention to how principals navigate competing demands while pursuing social justice goals.

### Limitations

As this study relies on self-reported data, participants’ accounts may be shaped by institutional and professional considerations, including confidentiality and reputational concerns. Accordingly, the findings reflect principals’ perspectives on their leadership enactment rather than exhaustive accounts of all aspects of social justice work within their schools. Also, the study draws on a small number of schools identified as exemplary in their engagement with social justice initiatives, and the findings may not be transferable to all school contexts. Future research could examine how these modes of leadership involvement operate in schools with fewer institutional supports or in systems characterized by more centralized policy control. Longitudinal research may also offer insight into how social justice initiatives evolve or change over time, particularly during leadership transitions.

In conclusion, this study suggests that social justice leadership in schools is best understood as a set of enacted leadership practices shaped by context, risk, and institutional constraint. By shifting attention from what principals *ought* to do toward how they engage in equity-oriented work in practice, this research contributes to a more grounded understanding of school leadership for social justice.
